# Influence of Heat Treatment on Surface, Structural and Optical Properties of Nickel and Copper Phthalocyanines Thin Films

**DOI:** 10.3390/ijms231911055

**Published:** 2022-09-21

**Authors:** Pawel Popielarski, Lidia Mosińska, Lukasz Skowronski, Robert Szczesny, Viviana Figà, Mieczyslaw Naparty, Beata Derkowska-Zielinska

**Affiliations:** 1 Institute of Physics, Kazimierz Wielki University in Bydgoszcz, Powstańców Wielkopolskich Str. 2, 85-090 Bydgoszcz, Poland; 2 Institute of Mathematics and Physics, Bydgoszcz University of Science and Technology, S. Kaliskiego 7, 85-796 Bydgoszcz, Poland; 3 Faculty of Chemistry, Nicolaus Copernicus University in Torun, Gagarina 7, 87-100 Torun, Poland; 4 Dipartimento di Scienze e Tecnologie Biologiche, Chimiche e Farmaceutiche, Università di Palermo, Viale delle Scienze, Parco d’Orleans II, 90128 Palermo, Italy; 5 Institute of Physics, Faculty of Physics, Astronomy and Informatics, Nicolaus Copernicus University in Torun, Grudziadzka 5, 87-100 Torun, Poland

**Keywords:** CuPc, NiPc, Raman, spectroscopic ellipsometry, refractive index, AFM, cyclic voltammetry

## Abstract

The work presents the effect of annealing on the change of polycrystalline α and β phases of copper and nickel phthalocyanines. We have found that this process has a great influence on the optical properties of the vapor-deposited layers. The performed measurements showed that for various forms of MPc, the values of the refractive index and the extinction coefficient increased, and consequently, so did the absorption coefficient. The AFM images taken showed that the values before and after heating are morphologically different. Raman measurements showed that the band at about 1526 cm^−1^ (B1g symmetry) has higher intensity for the α form than for the β form. The intensity of this band is related to changing the form of phthalocyanine from α to β. Our measurements have shown that by changing the annealing temperature of the layers, we change their optical properties. As a consequence, we change their optoelectronic parameters, adjusting them to the requirements of new optoelectronic devices, such as solar cells, sensors, displays and OLEDs.

## 1. Introduction

Organic materials used as thin layers are becoming increasingly important in the production of new devices for optoelectronics [1]. Phthalocyanines (Pcs) are an example of organometallic compounds, which are characterized by high chemical stability [2]. Currently, these materials are widely used in the production of devices such as organic light-emitting diodes (OLEDs), organic field effect transistors (OFET), organic photovoltaic cells (OPV) [3,4], gas sensors, biosensors [5,6], semiconductor connectors, memory, and other optoelectronic devices [7,8,9,10,11,12,13,14].

In recent years, there has been seen a great deal of interest in phthalocyanines with different metals as the central atom (called metallophthalocyanines—MPcs). Knowledge of a given phthalocyanine crystal structure provides the opportunity to find specific applications. The proper selection of the MPc thin layer orientation with a combination of determining the morphology and polymorphism of the obtained thin film structure, plays a significant role in optical and electrical properties [15,16,17,18,19,20]. There are many methods of depositing thin layers of phthalocyanine, which are divided into wet and dry. The selection of a specific method results in obtaining different physical properties of the obtained layers. Wet methods include drop-casting, dip-coating, spin-casting and spray-coating, whereas dry methods require more advanced equipment because the entire process takes place in a vacuum. The most frequently chosen methods of producing layers using the dry method are sputtering and physical vapor deposition.

In the case of phthalocyanines, we can distinguish several polymorphic phases, which are directly linked to the crystal structure. MPcs films exist in several molecular forms that range from amorphous to highly crystalline. However, it should be mentioned that the α and β forms are the most popular and stable forms at room temperature, which differ in the size of the tilt angle of the molecule within the columns and arrangement of the common columns in the crystalline structure. Moreover, the crystal structure is closely related to the deposition process (i.e., method, technology, evaporation rate) as well as the type, quality, orientation and temperature of the substrate surface. The heating of the obtained MPc layers, after the evaporation process, also affects their crystalline form [21,22,23,24,25,26]. Thus, it can be written that the deposition of layers by different methods and on different substrates determines different crystal structures of MPcs. Due to these conditions, MPcs thin layers have different physicochemical properties.

In this paper, we show how the process of thermal evaporation can determine the different crystalline forms of metallophthalocyanines and consequently how this process influences their optical properties as well as structural and surface properties. We compared the experimental results of less known nickel phthalocyanine (NiPc) with copper phthalocyanine (CuPc) [27,28]. We focused our studies on these MPcs because they have a more complex electronic structure since the 3d-like metal orbital lies between the HOMO and LUMO Pc. The optical properties of the α and β forms of CuPc and NiPc were determined using spectroscopic ellipsometry (SE), whereas the structural and surface properties were analyzed by AFM and Raman spectroscopy. Additionally, cyclic voltammetry (CV) measurements have been performed.

The long-range aim of this study is to learn how the thermal evaporation and substituting of different metal atoms into the ring of the phthalocyanines correlate with the surface, structural and optical properties, and how to enhance the properties by controlling the molecular structures so that these layers can be used in solar cells, sensors, displays and OLEDs.

## 2. Results and Discussion

### 2.1. AFM

**Figure 1:**
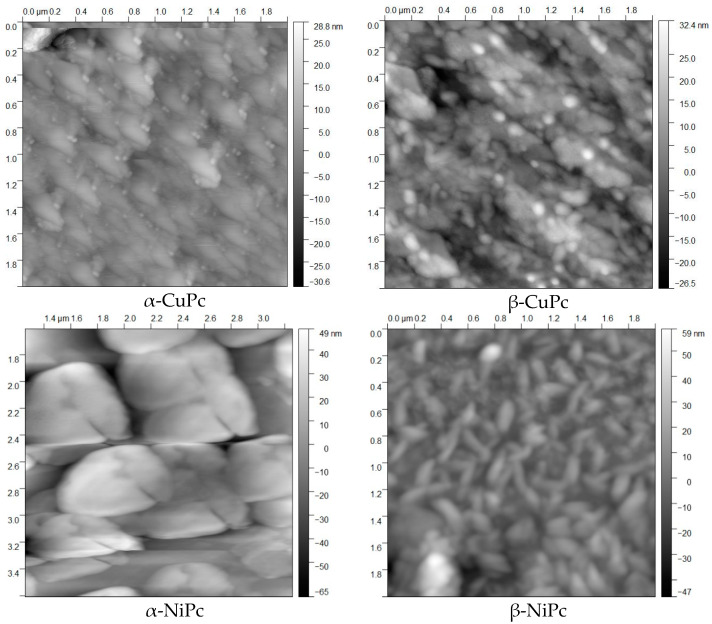
Surface topography of the α and β forms of CuPc and NiPc thin films obtained from AFM (2 × 2 μm^2^).

AFM measurements were used to compare morphology, surface and roughness of CuPc and NiPc thin films before and after annealing at 473 K. The results obtained for NiPc and CuPc samples before and after annealing are shown in Figure 1. To analyze and characterize the surface, two basic parameters such as root mean square (RMS) roughness and average grain size were used. It is visible that the surfaces of MPcs thin films before and after annealing at 473 K are different. This behavior is caused by obtaining a different crystalline form after the annealing of MPcs thin films at 473 K. It means that MPcs have the α form before annealing and the β form after annealing. The RMS roughness for the alpha and beta forms of NiPc were estimated to be 2 nm and 3 nm, respectively. The average grain size for each form was 40 nm and 50 nm, respectively. In the case of CuPc thin films, we observed similar values. The roughness was 4 nm and 5 nm for the sample before and after annealing, respectively, and the grain size was 40 nm and 50 nm. The measurement results are presented in Figure 1.

Therefore, it shows that the annealing process directly affects the homogeneity of the structure. In the case of unannealed samples, despite the larger grain size, the entire surface is homogeneous but has an aggregate and flake-like structure. The structures annealed at the temperature of 473 K have a slightly lower roughness parameter. A similar phenomenon was also observed for the deposition of thin films on substrates that were heated to temperatures above 400 K.

### 2.2. Raman

**Figure 2:**
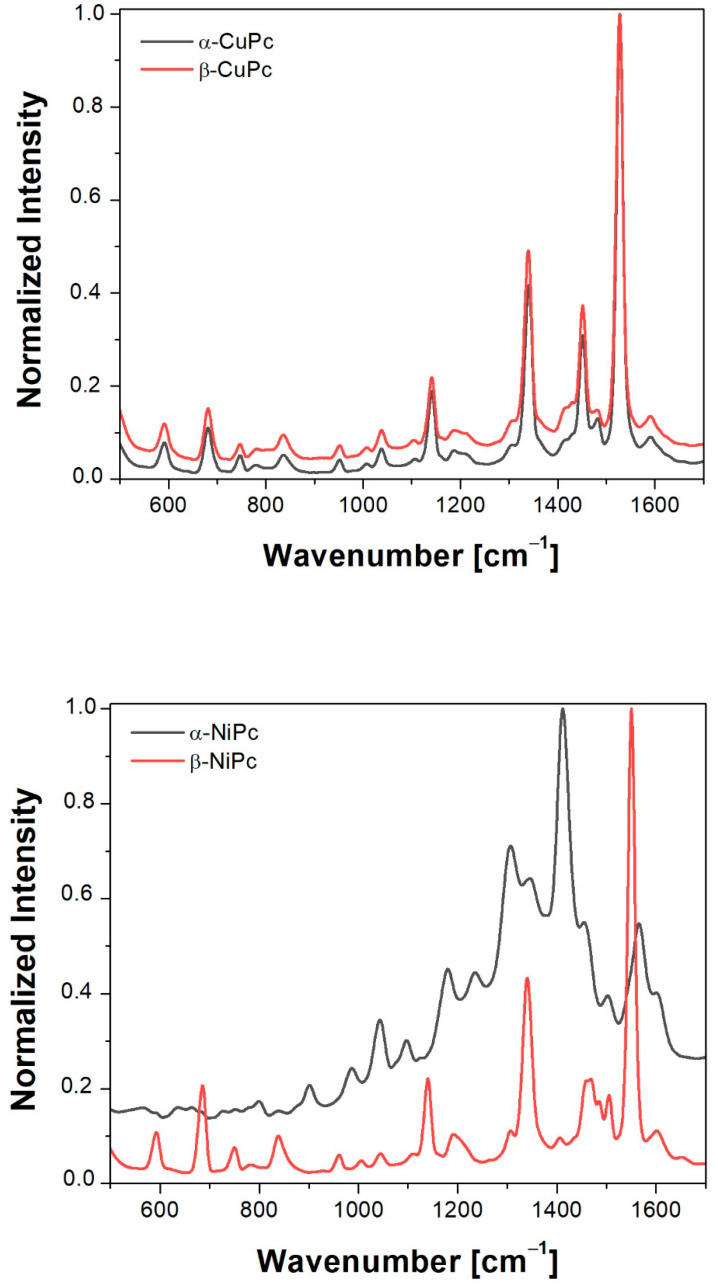
Normalized Raman spectra of the α and β forms of NiPc and CuPc thin films.

Figure 2 shows the normalized Raman unpolarized spectra of thin layers of copper and nickel phthalocyanines obtained by thermal evaporation in a vacuum. Normalization was completed to the second largest peak, which is shown in Figure S1 (in Supplementary Materials). These spectra were measured for CuPc and NiPc thin films before annealing and after their annealing at 473 K. In the CuPc spectra, we can observe that the band at about 1526 cm^−1^ (B1g symmetry) has higher intensity for the α form than for the β one. In the case of nickel phthalocyanine, compared with CuPc, the position of this band changes (it is located around 1551 cm^−1^) and the intensity of this band is also higher for the α form than for the β form. It should be noted that the position of this band is characteristic for phthalocyanines and is related to the displacement of C-N-C bridge bonds of the phthalocyanine macrocycle [29]. This relationship allows determining what metal ion is located in the center of the molecule. Therefore, comparing the intensities of individual spectra, it can be concluded that thin films annealed at the temperature of 473 K have lower intensity compared to unannealed samples. This behavior is related to a polymorphic change of form. The transformation, taking place in the annealing process, changes the angle of the molecule in relation to the substrate, which is directly visible in the Raman spectra. The peak positions and the assigned vibration modes are summarized in Table 1.

### 2.3. Spectroscopic Ellipsometry Measurements

**Figure 3:**
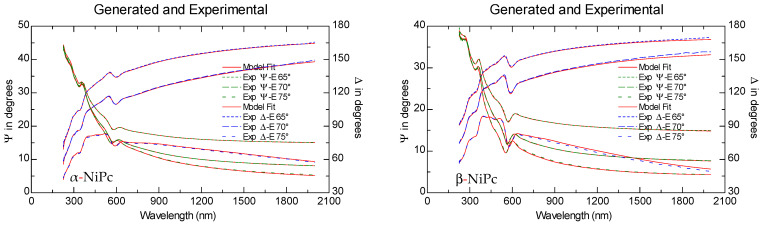
Experimental Ψ and Δ azimuths for three angles of incidence (65°, 70° and 75°) for the α and β forms of NiPc thin films.

The experimental ellipsometric azimuths (Ψ and Δ) and the adjustments of the obtained data from the optical model, for α and β forms of NiPc thin films deposited on n-type Si substrates with (100) orientation, are shown in Figure 3. The four-medium optical model of a sample (Si\native SiO_2_\MPcs\ambient) was used to determine both the thickness of the MPcs film and optical constants. The fit of the model is well suited to the experimental results. The reduced mean squared error was used to estimate the quality of the fit ($\chi^{2}$) [30,31]:

(1) $$\chi^{2}=\frac{1}{N\;-\;P}\sum_{j}\left(\frac{{\left(\Psi_{j}^{mod}\;-\;\Psi_{j}^{exp}\right)}^{2}}{\sigma_{\Psi j}^{2}}+\frac{{\left(\Delta_{j}^{mod}\;-\;\Delta_{j}^{exp}\right)}^{2}}{\sigma_{\Delta j}^{2}}\right)$$

In Equation (1), *N* and *P* are the total number of data points and the number of fitted model parameters, respectively. The quantities $\Psi_{j}$ and $\Delta_{j}$ are experimented (’exp’) or obtained from model (’mod’) ellipsometric angles. Quantities $\sigma_{\Psi j}$ and $\sigma_{\Delta j}$ are standard deviations for measured Ψ and Δ azimuths. The value of $\chi^{2}$ for the fits is established to be in the range from 2.4 to 8.5 (see Table 2). Based on these data, the extinction coefficients (*κ*) and the refractive indices (*n*) of the studied MPcs were determined.

**Figure 4:**
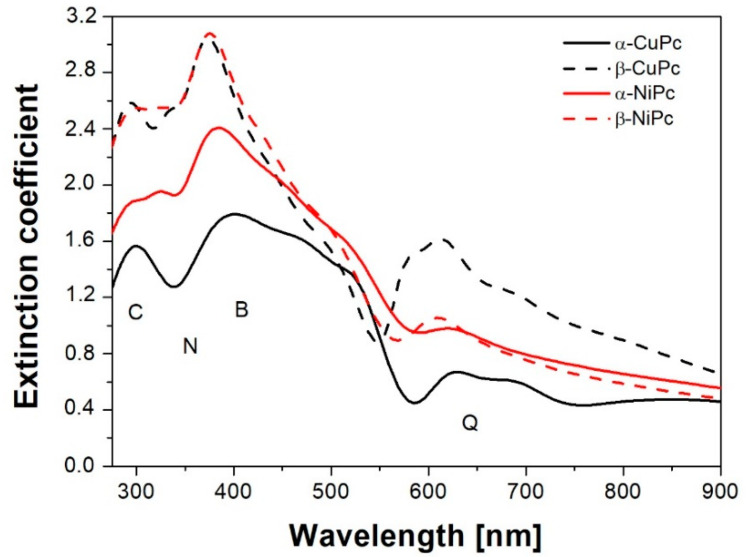
The extinction coefficient of the α and β forms of NiPc and CuPc thin films as a function of photon energy.

Figure 4 presents the extinction coefficient (*κ*) of the studied MPcs extracted from SE measurements. The shape of the *κ* spectra was parameterized using Gaussian oscillators, while the values of the interband transitions energy were determined from SE and are summarized in Table 2. Figure 4 illustrates four bands formed under the influence of molecular orbitals in the aromatic 18π electron system and overlapping orbitals bonding to the central metal atom. It should be mentioned that phthalocyanines containing certain transition metals (NiPc, CuPc) have more complex electronic structures because the metal 3d-like orbital lies between the HOMO and LUMO Pc. As a result of that, the spectra of these compounds can contain extra features arising from charge transfer transitions [32].

The first band in the range of 250–300 nm is called the C band and is due to d–π* transitions, which imply a broader d-band. Next, there is the N band that arises from the presence of the d-band associated with the central metal atom and resulting in the d–π* transitions, which have been attributed to the charge transfer transition from the sP_z_ mixing orbital to the electron system of the macrocyclic ring of the phthalocyanine. It should be noticed that the N band is more visible for NiPc compared to CuPc. In the range from 350 to 500 nm, we observe a direct electron transition from the π to π* orbitals. The observed intense transitions, called the Soret band (B band), give the edge of absorption for studied phthalocyanines in α and β forms [32,33]. The last Q-band is assigned to the first π–π* transitions on the phthalocyanine macrocycle. This band is split into two bands (Davidov splitting). These transitions and shifts are characteristic for phthalocyanines in crystal form, depending on the sample before and after annealing [27,34,35,36].

$\chi^{2}$ From Figure 4, we can note the difference in the shape of the absorption spectra of NiPc and CuPc. This feature may depend on the size of the phthalocyanine cavity and the symmetry of the molecule, which determine the state energies and the oscillator strength values. Metallic copper has an ion size similar to the cavity size of phthalocyanine, so it is accommodated in the cavity without any contraction or expansion of the ring and represents the phthalocyanine ring in its equilibrium state with a cavity diameter of 3.87 Å [29]. As a result, its structure is planar and possess approximately D_4h_ symmetry. In contrast, nickel phthalocyanine has a smaller cavity with a diameter of 3.66 Å; hence, its structure exhibits a contraction of the ring. Therefore, the four isoindole groups are pulled in toward the nickel to accommodate the smaller metal ion. This gives a smaller cavity diameter but also has an effect on the C-N-C bridge bonds, which are significantly lengthened compared to other phthalocyanine structures by around 0.05 Å, and the angle of the C-N-C bond is reduced by around 4°. Thus, to accommodate this small metal ion, a considerable degree of ring deformation takes place [29].

From Figure 4 and Table 2, it can be seen that the metal ion plays a crucial role in determining the shape and positions of particular bands for studied MPcs thin films. It is caused by different degrees of interaction between the metal ion and the phthalocyanine π system, which can depend on the number of electrons in the outer shell of the central metal [37]. Moreover, the surface morphology also depends on the metal substitution, and it can have a significant influence on the observed extinction coefficient. Additionally, it was found that heat treatment of the surfaces of the produced samples influences the shape of the extinction coefficient. It is also noticeable that the value of the extinction coefficient increases for CuPc annealed at 473 K in the whole of the measured region, which can be a result of the reduction in grains deposited on the substrate by subjecting the sample to the heating process [33,38]. However, for NiPc, this increase is visible only in the range of 250–450 nm.

**Figure 5:**
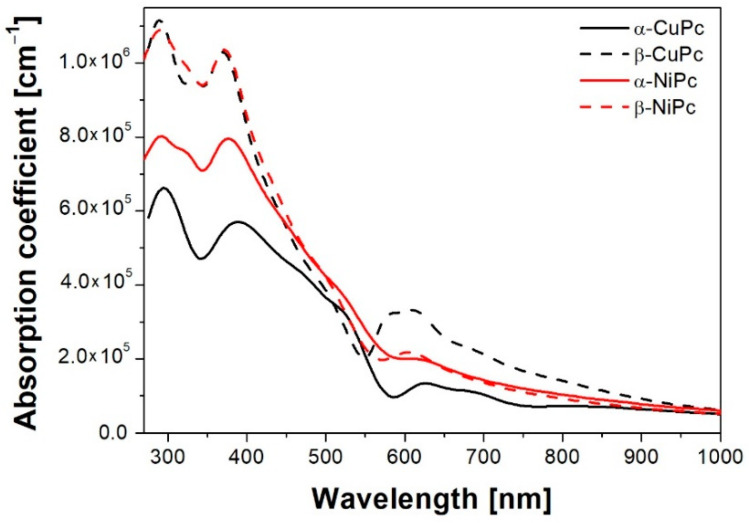
Absorption coefficient of the α and β forms of NiPc and CuPc thin films.

From the extinction coefficient data, the absorption coefficient was determined using a well-known equation $\alpha =\frac{4\pi \kappa }{\lambda }$ and is shown in Figure 5. It can be seen that in general, the values of the absorption coefficient are higher for the β form of the studied MPcs than for the α form. For CuPc in the range of 250–400 nm, this difference is almost twice.

**Figure 6:**
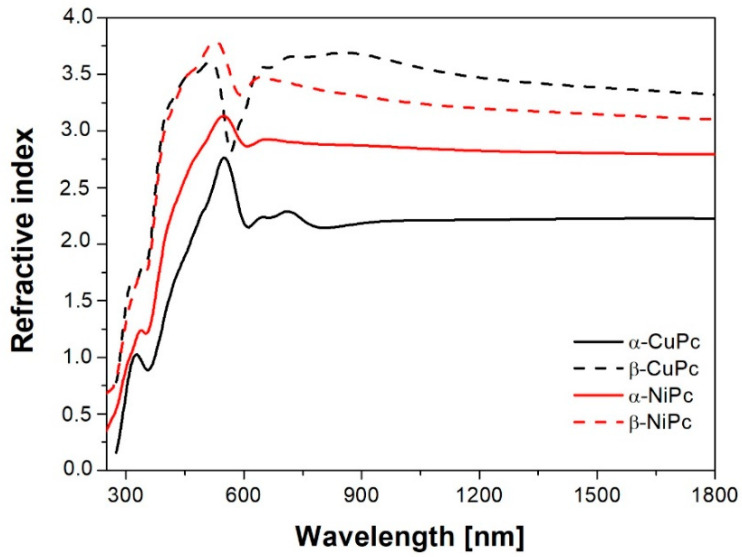
The refractive index of the α and β forms of NiPc and CuPc thin layers as a function of photon energy.

Figure 6 shows the refractive indices (*n*) of the α and β forms of NiPc and CuPc thin layers, which are the basic properties of the material used in the design of optoelectronic devices. One can see that an anomalous dispersion in the absorption region and normal dispersion in the transparent area are visible. The values of refractive indices for α-NiPc and β-NiPc are close to each other, especially in the region for *λ* > 800 nm. However, the *n* is higher for the β form of NiPc compared to the α form. In the case of CuPc, greater differences between the values of refractive index for α and β forms are apparent. The value of *n* for the β form increased almost one and a half times compared with the α form.

### 2.4. Electrochemical Investigation

**Figure 7:**
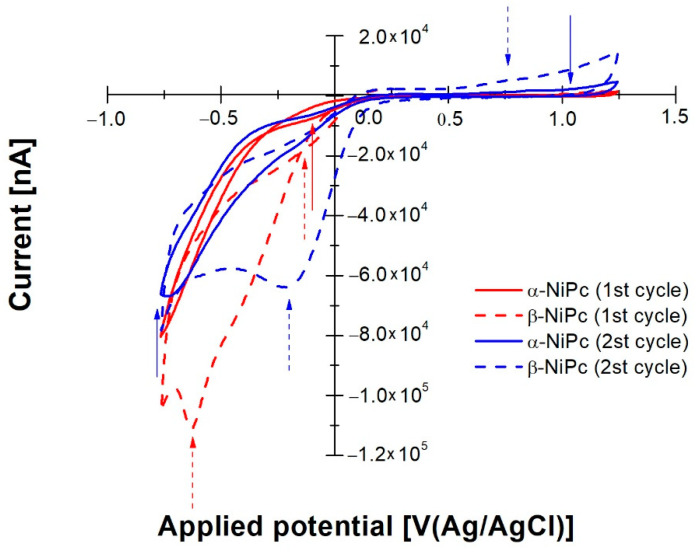
Superimposition of cyclic voltammograms of NiPc in α and β forms for first and second cycles.

Thin films of NiPc and CuPc in α and β forms, deposited onto FTO/glass electrodes, were characterized by means of cyclic voltammetry (CV) starting from the open circuit values up to 1.24 V/(Ag/AgCl). The reverse scan explored the cathodic branch down to −0.8 V/(Ag/AgCl). Figure 7 shows the superimposition of cyclic voltammograms of NiPc in α and β forms. In particular, Figure 7 indicates the first and the second cycle of the two films.

For both of them, the anodic branch of the first cycle does not evidence any redox activity of these films. The current increase at around +0.9 V/(Ag/AgCl) can be assigned to oxygen evolution. By inverting the scan direction, NiPc α and β forms show cathodic activity. The α-NiPc shows a reduction onset at −0.22 V/(Ag/AgCl), while the β form shows one at −0.053 V/(Ag/AgCl). The second scan (see Figure 7) shows a shoulder at around −0.16 V and a reduction peak at −0.21 V/(Ag/AgCl) for α-NiPc and β-NiPc, respectively. According to Ding et al. [39], the cathodic processes at −0.16 V/(Ag/AgCl) and −0.21 V/(Ag/AgCl) can be ascribed to the oxygen reduction, which is more evident in the case of NiPc in the β form. The anodic peaks of the second cycle located at 0.77 V/(Ag/AgCl) and 0.88 V/(Ag/AgCl) can be assigned to the interaction between the phthalocyanine macro-ring and the central metal [40].

It is known that the reduction onset is related to the electron affinity according to the following equation:

(2) $$EA=E_{onset}^{red}+4.5$$

where $E_{onset}^{red}$ is the reduction potential onset with respect to NHE [41].

NiPc films annealed at different temperatures show electron affinities (EA) of 4.485 eV and 4.652 eV for α and β forms, respectively.

**Figure 8:**
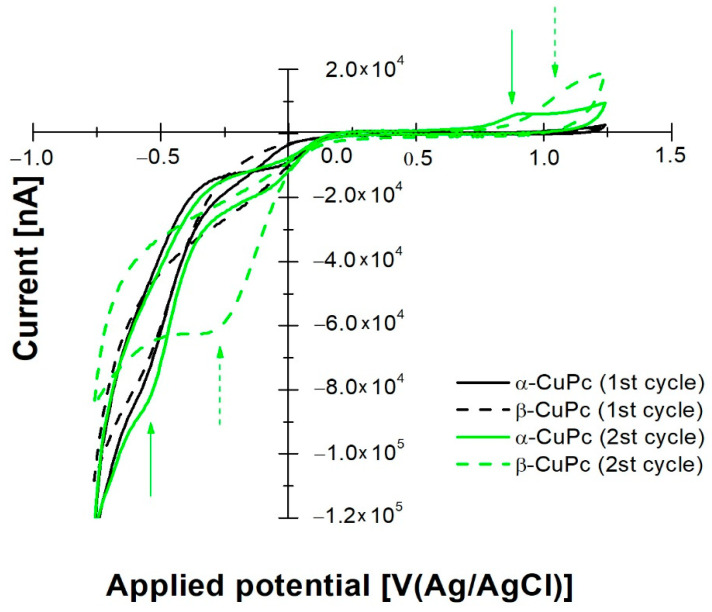
Superimposition of cyclic voltammograms of CuPc in α and β forms for first and second cycles.

Figure 8 shows the first and the second cycles of CV of CuPc films differently annealed.

In the case of copper phthalocyanines, the anodic branch of first cycle does not show any process connected to the film itself. Only an increasing of the anodic current associated to the oxygen evolution reaction is observed starting from 0.9 V/(Ag/AgCl). By inverting the potential scan, both α and β forms show cathodic activity. In particular, they show reduction onsets at −0.19 V/(Ag/AgCl) and −0.25 V/(Ag/AgCl), respectively. According to Equation (2), electron affinity values of 4.515 eV and 4.455 eV were calculated.

The second cycles (Figure 8) display anodic activities of CuPc films with a peak at 0.88 V/(Ag/AgCl) in the case of the α form and an anodic wave starting from 0.92 V/(Ag/AgCl) in the case of the β form. The reverse scan shows two reduction peaks at −0.56 V/(Ag/AgCl) and −0.29 V/(Ag/AgCl) for the α and β forms, respectively, which can be associated to the anodic ones, suggesting that quasi-reversible processes take place in CuPc films.

Generally, by comparing the electrochemical responses, it is evident that in both metal–phthalocyanine (NiPc and CuPc) compounds, the annealing temperature affects the redox activity of the films. Higher EAs value are calculated in the case of β forms.

## 3. Materials and Methods

### 3.1. Metallophthalocyanines

**Figure 9:**
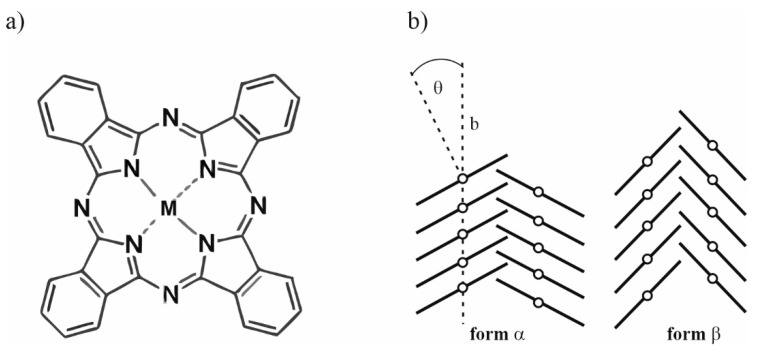
(**a**) Molecular structure of MPc (where M = Cu, Ni); (**b**) α and β forms of phthalocyanine, where θ is the angle between the *z* axis of the molecule and the *b* axis of the crystal structure (where Bartolomé et al. [42] have found that θ is ≈26.5° for α-NiPc and α-CuPc, and θ is ≈46.5° for β-NiPc and β-CuPc).

Metallophthalocyanines (MPcs) are one of the most important metalloorganic materials used in physics and chemistry. The molecular structure of MPc is shown in Figure 9a. In the structure of phthalocyanines, we can distinguish four isoindole rings, which are connected to each other by an azamethene bridge. Their structure resembles porphyrins, in which we can distinguish four pyrrole rings connected with each other by means of methine bridges (=C-). Two hydrogen atoms or a metal cation may be attached to the center of the phthalocyanine ligand. We can assume that the coordination compound consists of an electron acceptor (a cation or a metal atom), an energetically low empty orbital, and an electron donor. Its function is performed by a ligand because it has unbound, lone pairs of electrons and does not have low-lying empty orbitals energetically. Currently, phthalocyanines in their structure may contain non-transition and transition metals. We can distinguish about 70 elements forming complexes with phthalocyanine.

One of the most interesting properties of phthalocyanines is their polymorphism. We can observe this in the crystal and thin layer. Thin layers of phthalocyanines deposited on the various substrates can range from amorphous to crystalline. Molecules are usually arranged in columnar piles in crystalline form as they grow on the substrate. The best-studied phthalocyanine in this respect is CuPc, where nine different polymorphs are known [43]. However, the most interesting and permanent forms are α and β. Figure 1 shows the differences between these two forms of copper phthalocyanine.

Taking into account the differences between the polycrystalline α and β phases, special attention should be paid to the size of the crystallites. In the case of the α form, the crystallites are about 100 Å, while for the β form, they are much larger. The thickness of the obtained layer also affects the grain size, i.e., with increasing thickness, an increase in crystallites is observed [44], which are situated perpendicular to the substrate, and the layer has a poorly packed structure [45]. It is possible to obtain an amorphous phthalocyanine layer by the thermal evaporation method. However, for this purpose, the sublimation process is carried out at a pressure of 0.0013 Pa, and the temperature of the substrate should not exceed 100 K. It is possible to change the amorphous to the crystalline phase. In this case, in order to obtain the α form, it is necessary to anneal the layers at a temperature of 353 K, whereas to obtain the β form, it is necessary to heat the α form at temperatures above 473 K [46]. It should be noted that by changing the polymorphic form, the optical properties also change.

### 3.2. Preparation of Thin Films

Thin layers of nickel and copper phthalocyanines were obtained by thermal evaporation in a vacuum (*p* = 2 × 10^−6^ Torr). Quartz and n-type silicon with (100) orientation were used as substrates. Each of them was properly cleaned with acetone, ethanol and finally rinsed in deionized water. Afterwards, the substrate and material were placed in a thermal evaporation chamber and initially heated to remove water vapor; then, they were brought to the evaporation temperature (393 K for CuPc and NiPc). The steaming process was continued until the required layer thickness was achieved. The deposition rate was 0.1 nm/s. From this procedure, the thin films of α phase with a thickness of about 30–40 nm were obtained. The β form was formed as a result of eight hours of annealing the α form at 473 K [21].

### 3.3. Experimental Methods

The Innova (Bruker) measuring system in a tapping mode was used to take AFM images (selected area 2 × 2 μm^2^). Two-dimensional (2D) images conversion software was used to estimate the average size of aggregates and the RMS roughness parameter. Measurements were made at room temperature.

The Raman spectra were recorded using the Raman spectrometer (Senterra by Bruker Optik) in the spectral range of 500–1700 cm^−1^, where a laser of 532 nm (10 mW) was used as a source of excitation [47]. The laser beam was tightly focused on the sample surface through a Leica 20× microscope objective. To prevent any damages of the sample, an excitation power was fixed at 5 mW.

We used the spectroscopic ellipsometry (SE) in order to characterize the optical properties such as refractive index (*n*) and extinction coefficient (*κ*) of the studied films deposited on n-type Si substrates with (100) orientation. The SE measurements were made using a V-VASE ellipsometer (J.A. Woollam Co., Inc., Lincoln, NE, USA) in the range of 250–2000 nm for three angles of incidence (65°, 70°, 75°). The complex dielectric functions ($\tilde{\varepsilon }=\varepsilon_{1}+i\varepsilon_{2}$, where $\varepsilon_{1}=n^{2}-\kappa^{2}$ and $\varepsilon_{2}=2n\kappa$ are the real and imaginary parts, *n* and $\kappa =\frac{\alpha^{\prime }\lambda }{4\pi }$ are the refractive index and extinction coefficient, *α’* is the absorption coefficient, *λ* is wavelength) for the investigated thin films were calculated directly from the ellipsometric data using the WVASE32 software. These optical constants were parameterized using Gauss-shape dispersion relation in the absorption regime.

The electrochemical properties of CuPc and NiPc in α and β forms were investigated by cyclic voltammetry (CV). Thin films of CuPc and NiPc α and β were deposited on ITO/quartz substrates and used as working electrodes in a three-electrode cell. Graphite wire and Ag/AgCl (3.0 M KCl) were used as counter and reference electrodes, respectively. For avoiding the dissolution of metal phthalocyanines films in common organic solvents, the voltammograms were recorded in 0.1 M KCl (Sigma-Aldrich for molecular biology, ≥99.0%) aqueous solution. Cyclic voltammograms were acquired by using an Ivium Vertex Potentiostat/Galvanostat at a scan rate of 200 mVs^−1^ starting from the open circuit potential in a potential window between 1.24 and −0.8 V/(Ag/AgCl). All the measurements were carried out at room temperature in an aerated solution.

## 4. Conclusions

The influence of heat treatment of nickel and copper phthalocyanines (NiPc and CuPc), which leads to the transition of phthalocyanine from α to β and the rearrangement of the molecular structure, on their structural and optical properties in terms of use in OLED technology is presented. We found that this change influenced the physical properties of the studied organic materials. We have shown that the physical properties of the studied NiPc and CuPc thin layers are closely related to the polymorphic phase of each of the phthalocyanines.

In our research, we observed a change in the intensity of Raman spectra for the samples before and after annealing at 473 K. It was also found that the heat treatment of the studied MPcs increased the values of the refractive index and the extinction coefficient as well as the absorption coefficient. Moreover, it was shown that the values of the extinction coefficient, the absorption coefficient and the refractive index are higher for α-NiPc than for α-CuPc. In contrast, from electrochemical study, we found that the electron affinity is the highest for β-NiPc.

We noticed that the obtained results show the stability of NiPc and CuPc thin layers after the thermal evaporation process and annealing.

Our results show that the produced layers are suitable for use in solar cells, sensors, displays and OLEDs.

## Figures and Tables

**Table 1:** Peak positions (*PP*), peak intensity (*PI*) and assigned vibration modes for NiPc and CuPc thin films.

α-CuPc		β-CuPc		α-NiPc		β-NiPc		
*PP* (cm^−1^)	*PI* (a.u.)	*PP* (cm^−1^)	*PI* (a.u.)	*PP* (cm^−1^)	*PI* (a.u.)	*PP* (cm^−1^)	*PI* (a.u.)	Assigned Symmetry of Vibration
588.9	2798	593.7	2505	593.7	6532	595.1	3792	B_1g_ C–N vib.
682.35	3462	682.35	2956	688.55	11,792	688.5	6912	A_1g_ C–C vib.
748.74	2253	746.92	1827	749.84	4674	749.84	2816	B_1g_ C–C vib.
837.39	2095	836	2236	839.4	6219	838.8	3527	Out-of-plane ring deformation
1005	1629	1008.4	3364	1004.4	3442	1007.7	2368	A_1g_
1039.85	2541	1040.9	2649	1044.6	4214	1047.5	2550	C–H bend
1105	1966	1107.3	1693	1108.8	4214	1108.3	2816	A_1g_ C–H bend
1140	5957	1142	3994	1142.7	12,565	1142.7	7092	A_1g_ Pyrrole ring deformation
1190.86	2382	1194	2327	1197.9	6532	1194.8	3707	B_2g_
1338.9	12,276	1340	8220	1341.9	23,214	1341.9	13,427	A_1g_ pyrrole stretch
1453.9	9196	1452.4	6392	1455.35	12,252	1461.5	7188	B_2g_
1482	4204	1486	3091	1473.9	12,565	1486	5670	
1526	28,419	1529	16,150	1555	52,109	1553.4	30,267	B_2g_ C–N vib.
1591	3046	1591	2801	1604.1	6992	1602.7	3887	

**Table 2:** The values of the thicknesses (d), the reduced mean squared error ($\chi^{2}$) and energies of absorption bands (E) of α and β forms of NiPc and CuPc thin layers.

	α-NiPc	β-NiPc	α-CuPc	β-CuPc
d (nm)	37 ± 1	45 ± 1	30 ± 1	86 ± 1
$\chi^{2}$(–)	2.4	3.0	5.7	8.5
E_1_ (eV)	5.17 ± 0.04	5.39 ± 0.01	3.93 ± 0.01	4.66 ± 0.63
E_2_ (eV)	4.60 ± 0.16	4.05 ± 0.09	2.90 ± 0.02	4.02 ± 0.07
E_3_ (eV)	4.03 ± 0.05	4.00 ± 0.97	2.46 ± 0.03	3.65 ± 0.01
E_4_ (eV)	3.71 ± 0.02	3.64 ± 0.03	2.31 ± 0.01	3.26 ± 0.04
E_5_ (eV)	3.09 ± 0.08	3.23 ± 0.03	1.97 ± 0.01	2.81 ± 0.06
E_6_ (eV)	2.60 ± 0.05	2.83 ± 0.04	1.80 ± 0.01	2.41 ± 0.01
E_7_ (eV)	2.31 ± 0.02	2.41 ± 0.04	1.55 ± 0.03	2.15 ± 0.01
E_8_ (eV)	2.14 ± 0.01	2.04 ± 0.01	1.08 ± 0.52	1.83 ± 0.05
E_9_ (eV)	2.00 ± 0.02	1.90 ± 0.12	-	1.25 ± 0.32
E_10_ (eV)	1.91 ± 0.31	1.79 ± 0.34	-	-
E_11_ (eV)	1.74 ± 0.38	1.42 ± 0.74	-	-
E_12_ (eV)	1.28 ± 0.96	1.40 ± 0.01	-	-

## Data Availability

Data supporting the results of this study are available from the appropriate author upon reasonable request.

## Supplementary Materials

The following supporting information can be downloaded at: https://www.mdpi.com/article/10.3390/ijms231911055/s1.
