# Supplementary figures and images for: Influence of Heat Treatment on Surface, Structural and Optical Properties of Nickel and Copper Phthalocyanines Thin Films
Source: Int J Mol Sci. 2022 Sep 21;23(19):11055. doi: 10.3390/ijms231911055 (PMC9569429; doi:10.3390/ijms231911055)

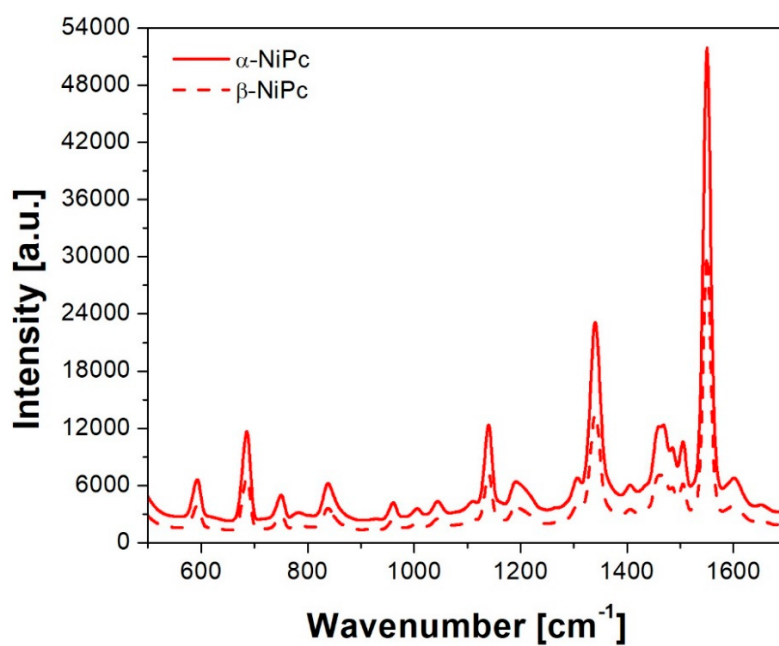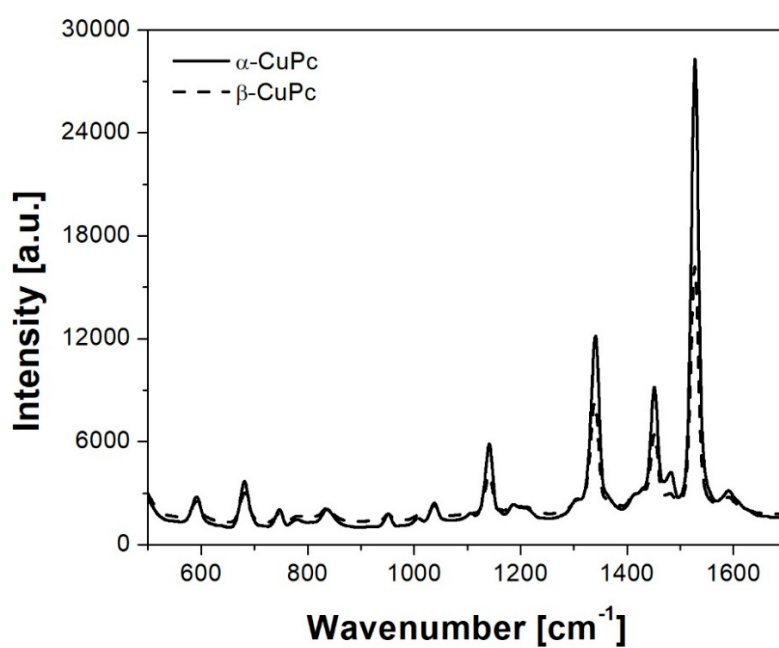

**Figure S1.** Raman spectra of the  $\alpha$  and  $\beta$  forms of NiPc and CuPc thin films.

Supplement: Supplementary file 1 [file ijms-23-11055-s001.zip › ijms-1895945-supplementary.pdf]
